# FcircSEC: An R Package for Full Length circRNA Sequence Extraction and Classification

**DOI:** 10.1155/2020/9084901

**Published:** 2020-05-28

**Authors:** Md. Tofazzal Hossain, Yin Peng, Shengzhong Feng, Yanjie Wei

**Affiliations:** ^1^Joint Engineering Research Center for Health Big Data Intelligent Analysis Technology, Center for High Performance Computing, Shenzhen Institutes of Advanced Technology, Chinese Academy of Sciences, Shenzhen, Guangdong Province, China 518055; ^2^University of Chinese Academy of Sciences, No. 19(A) Yuquan Road, Shijingshan District, Beijing, China 100049; ^3^Department of Pathology, The Shenzhen University School of Medicine, Shenzhen, Guangdong, China 518060

## Abstract

Circular RNAs (circRNAs) are formed by joining the 3′ and 5′ ends of RNA molecules. Identification of circRNAs is an important part of circRNA research. The circRNA prediction methods can predict the circRNAs with start and end positions in the chromosome but cannot identify the full-length circRNA sequences. We present an R package FcircSEC (Full Length circRNA Sequence Extraction and Classification) to extract the full-length circRNA sequences based on gene annotation and the output of any circRNA prediction tools whose output has a chromosome, start and end positions, and a strand for each circRNA. To validate FcircSEC, we have used three databases, circbase, circRNAdb, and plantcircbase. With information such as the chromosome and strand of each circRNA as the input, the identified sequences by FcircSEC are consistent with the databases. The novelty of FcircSEC is that it can take the output of state-of-the-art circRNA prediction tools as input and is applicable for human and other species. We also classify the circRNAs as exonic, intronic, and others. The R package FcircSEC is freely available.

## 1. Introduction

Circular RNAs (circRNAs) are formed by joining a downstream 3′ splice donor site and an upstream 5′ splice acceptor site in the primary transcript [1]. In most cases, circRNAs originate from exons close to the 5′ end of a protein coding gene and may consist of one or more exons. Furthermore, multiple circRNAs can be produced from a single gene. circRNAs are generated through several distinct mechanisms that rely on complementary sequences within flanking introns [2–4], exon skipping [4, 5], and exon-containing lariat precursors [6]. circRNAs were first discovered approximately 40 years ago and thought to be an RNA splicing error [7]. Until 2013, the researchers did not pay much attention in this area, but after publishing the paper [8], the circRNA research turned into a prominent field in scientific research. A significant amount of circRNAs is identified through the high-throughput RNA sequencing and bioinformatics analysis [9, 10]. In recent years, many types of circRNAs have been identified and found to be stable and abundant [2]. One of the important properties of circRNA is that they have tissue-specific expression. Several studies conclude that circRNAs are substantially enriched in brain tissues and the expression levels are dynamic during brain development of human and mice brain tissues [11–13]. circRNAs show differential expressions between primary ovarian tumors and metastatic tumors in ovarian carcinoma [14]. Some circRNAs also interact with RNA-binding proteins (RBPs) [15] although very little enrichment in binding sites of RBPs is found for circRNA sequences compared with those of its corresponding linear mRNA. The studies [8, 16, 17] reveal that circRNAs can bind to a few RNA-binding proteins (RBPs), such as Argonaute and MBL. circRNAs are conserved across different species and act as a microRNA (miRNA) sponge while miRNAs have oncogenic or tumor suppressor properties [18]. Although the function of most circRNAs is unknown, some functions of the circRNAs are known as miRNA sponges [8, 19, 20], protein translation templates [21–24], and regulation of gene expression [25–28]. Different studies suggest that circRNAs are important biomarkers for different cancers [29–31] and autoimmune diseases [32, 33], a potential noninvasive diagnosis for atherosclerosis [34], disorders of the central neural diseases [35], degenerative diseases [17], and cancers [10, 36].

Identification of circRNAs is a crucial step for circRNA research. A number of methods is available for the identification of circRNAs such as CIRI [37], circRNA_finder [38], DCC [39], find_circ [40], segemehl [41], CIRCexplorer [3], MapSplice [42], and UROBORUS [43]. Each of these methods can predict circRNAs and their position in the chromosome, but these methods cannot provide the full-length circRNA sequence. To infer/predict the function of the circRNAs, differential expression analysis and network analysis are very common, and full-length circRNA sequences are required. CIRI-full [44] can extract the full-length circRNA sequences from its own output of CIRI; however, the method fails for the unequal read lengths in a sample and does not accept the annotation file in gff format. FUCHS [45] does not provide a full-length circRNA sequence directly; only when using its output with additional software is it possible to obtain the full-length circRNA sequences. Besides, FUCHS is tested for the output of DCC only. Recently, a software tool circtools [46] has been published as a one-stop software solution for circRNA research which also uses the FUCHS module. Another method CircPrimer [47] can extract the full-length circRNA sequences although its main function is to design primers. CircPrimer cannot extract circRNA sequences other than for humans. The output of CIRI, find_circ, circRNA_finder, DCC, and segemehl gives three types of circRNAs (exonic, intronic, and intergenic). CIRCexplorer and MapSplice give two types (exonic and intronic) while UROBORUS gives only one type (exonic) of circRNAs in their output. Again, FUCHS, circtools, and CircPrimer cannot provide circRNA classification. A number of papers [48–50] classified their circRNAs as these five types: exonic, intronic, intergenic, sense overlapping, and antisense. Another paper [51] classified circRNA as exonic, intronic, intergenic, bidirectional/intragenic, and antisense. Our realization is that circRNA classification is not finished yet. The existence of exonic and intronic circRNA is supported by numerous biological experiments, but other types are rarely validated by PCR experiments. Therefore, we have classified the circRNAs as exonic, intronic, and others.

There are four available tools for extracting full-length circRNA sequences, CIRI-full, FUCHS, circtools, and CircPrimer. CIRI-full utilizes both BSJ (back-splice junction) and RO (reverse overlap) features to obtain full-length circRNA sequences. CIRI-full uses the output of CIRI, and RNA-seq data is needed to reconstruct the full-length sequence. The main limitation of CIRI-full is that it is not applicable if the sequencing read lengths are not equal for all reads in the RNA-seq data. Besides, CIRI-full does not accept the annotation file in gff format. FUCHS is developed to fully characterize candidate circRNA sequence utilizing all RNA-seq information from long reads (>150 bp). It is tested for the output of DCC only and not applicable for short reads. Besides, FUCHS cannot provide a full-length circRNA sequence directly. circtools is designed for RBP enrichment screenings and circRNA primer design, as well as circRNA sequence reconstruction. For circRNA sequence reconstruction, circtools utilizes the FUCHS module. The main function of CircPrimer is to design primers for circRNAs. Additionally, it can extract full-length circRNA sequences. It depends on the annotation information and is useful for human circRNAs only.

In this paper, we present an R package FcircSEC to extract directly the full-length circRNA sequences and to classify the circRNAs utilizing the output of circRNA prediction methods and the gene annotation information. We have followed the approach similar to CircPrimer in extracting circRNA sequences. Like CircPrimer, FcircSEC first selects the best transcript from the annotation file, then from the part of the transcript within the circRNA boundary, the introns are removed and finally combines all the exon sequences as a circRNA sequence. But our best transcript selection strategy (described in Materials and Methods) is different from CircPrimer. Even CircPrimer is applicable for human circRNAs only, but FcircSEC is useful for human and other species. FcircSEC only needs the output of the circRNA prediction tool along with the reference genome and the annotation file. The main advantage of FcircSEC is that it can use the output of many state-of-the-art circRNA prediction tools for extracting the actual sequence (with information on chromosome, circRNA start and end position, and strand). As there are no tools for full-length circRNAs for the user of the circRNA prediction tools other than CIRI and DCC, FcircSEC can be a good choice for them.

## 2. Materials and Methods

In our R package FcircSEC, from the gene annotation information of the reference genome, we extracted all transcripts and got the number of exons with their start and end positions for each transcript. Then, we selected the best transcript using the output of circRNA prediction methods. Finally, we extracted the full-length circRNA sequences from the selected best transcript. To check the validity of our package, we used human circRNAs from two popular databases circbase (http://circbase.org/) and circRNAdb (http://202.195.183.4:8000/circrnadb/circRNADb.php) and plant circRNAs from the plantcircbase (http://ibi.zju.edu.cn/plantcircbase/) database. The circRNA sequences obtained by FcircSEC were consistent with the databases.

**Figure 1:**
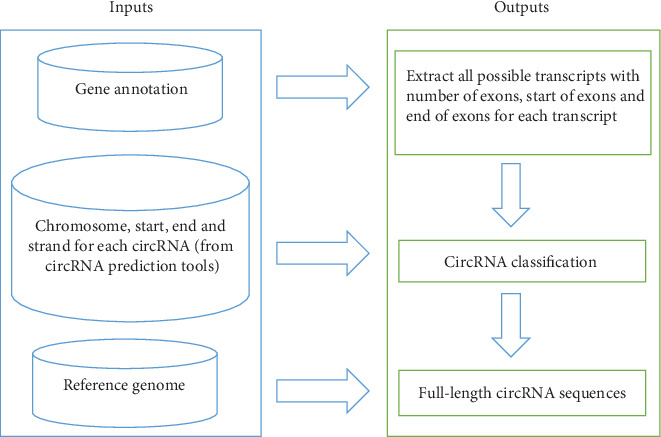
Workflow of the FcircSEC package. From the gene annotation file, a nine-column transcript data file is generated for all transcripts with the number of exons and the start and end of each exon. Using the transcript data and the output of the circRNA prediction tool, the circRNA classification is done. Using the circRNA classification information and the reference genome, the full-length circRNA sequences are extracted.

The package needs three input files: (1) the four types of information (chromosome name, start position, end position, and strand of the circRNAs) from the output of circRNA prediction tools, (2) the reference genome, and (3) the annotation file corresponding to the reference genome. Inputs (2) and (3) can be downloaded from UCSC, NCBI, or any other databases, and input (1) can be obtained from circRNA prediction tools like CIRI, find_circ, circRNA_finder, DCC, CIRCexplorer, segemehl, MapSplice, and UROBORUS whose outputs have the abovementioned four types of information. The genome versions used in our analysis for different species are given in Table 1. The flowchart of FcircSEC is provided in Figure 1.

### 2.1. Location-Oriented Classification of Circular RNA

**Figure 2:**
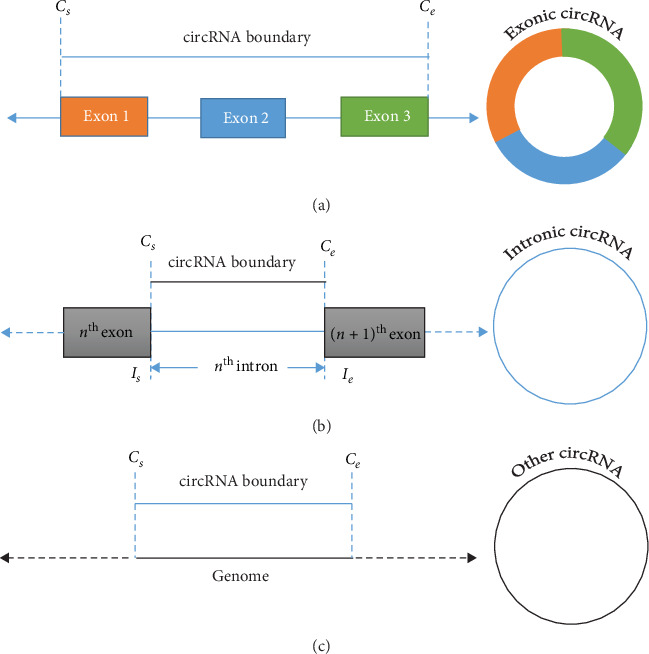
Location-oriented classification of circRNA. (a) The circRNA is exonic as it is formed of three exons (exons 1, 2, and 3). (b) The circRNA is intronic as it consists of one intron only. (c) The circRNA type is other as it belongs to neither exonic nor intronic.

We classified circRNAs as three types: exonic, intronic, and others. *Exonic*: if the circRNA is originated from one or more exons of the linear transcript and the transcript strand and the circRNA strand are same, then the circRNA is exonic. *Intronic*: if the circRNA is originated from an intron of the linear transcript, then the circRNA is intronic. *Other*: if the circRNA belongs to neither exonic nor intronic, we call it as other type. The three types of the circRNAs are shown in Figure 2.

### 2.2. Extraction of Transcript Information from the Gene Annotation File

In this step, the input was the gene annotation file of the reference genome. The annotation file has nine columns: seqname, source, feature, start, end, score, strand, frame, and attribute. To extract the transcript information from the annotation data, the following steps were followed:

*Step 1*: from the attribute column of the annotation file, extract the transcript name and the gene name

*Step 2*: for each unique transcript, count the number of exons and obtain the start position and end position of each exon

*Step 3*: subtract 1 from the start position of the exons

*Step 4*: make a 9-column text file with transcript name (ID), chromosome, transcript strand, transcript start, transcript end, number of exons in each transcript, start positions of exons, end positions of exons, and gene name

### 2.3. Selection of the Best Transcript

The inputs of this step were the transcript data obtained from the previous Section 2.2 and the four columns (chromosome, start position, end position, and strand of circRNAs) from the output of circRNA prediction methods. In the best transcript selection, we followed two strategies. We selected the transcript whose coordinates (an interval from transcript start to end) contained the circRNA boundary. If there were several such transcripts, we selected all of them. For all possible transcripts, we checked whether the circRNA start and end positions exactly matched or not with the start of the first exon and end of the last exon, respectively. If yes, we selected that transcript as best transcript which has the longest splice sequence (sequence of all combined exons). If not, we selected the transcripts having maximum number of exons and then selected the one having the maximum length.

**Figure 3:**
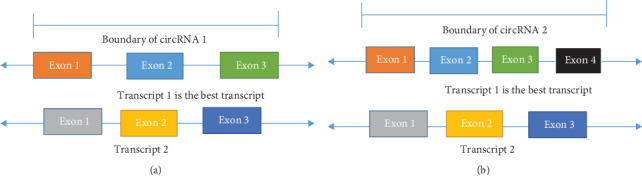
Best transcript selection. (a) There are two possible transcripts within the circRNA boundary. Transcript 1 is the best transcript as its start and end positions are exactly matched with those of the circRNA. (b) Within the circRNA boundary, there are two possible transcripts, and for both, the transcripts' start and end positions are not exactly matched with those of the circRNA. Transcript 1 is the best transcript as it has a larger number of exons than transcript 2.

Let *T* be all transcripts extracted from the gene annotation file and *O* be the output from the circRNA prediction tool. For *i*^th^ circRNA of *O*, all possible transcripts *T*_possible_ were selected containing the circRNA boundary (e.g., transcripts 1 and 2 for circRNA 1 in Figure 3(a)). Then, the best transcript was selected using case 1 and case 2.

**Case 1 .**

For any transcript from *T*_possible_, if the start position of the first exon and the end position of the last exon are exactly matched with the circRNA boundary (e.g., transcript 1 in Figure 3(a)), select that transcript. If more than one such type of transcript is selected, repeat the following steps until a single transcript is selected:

1. select the transcript having the maximum splice length (length of all combined exons)
2. select the transcript having the maximum transcript length
3. select the first one

**Case 2 .**

For all transcripts from *T*_possible_, if the start position of the first exon and the end position of the last exon are not exactly matched with the circRNA boundary (e.g., transcripts 1 and 2 in Figure 3(b)), select that transcript having the maximum number of exons within the boundary (e.g., transcript 1 in Figure 3(b)). If more than one such type of transcript is selected, repeat the following steps until a single transcript is selected:

1. select the transcript having the maximum transcript length
2. select the first one

### 2.4. Circular RNA Classification and Sequence Extraction

The inputs of this step were the best transcript obtained from the previous Section 2.3, the four columns of the outputs of the circRNA prediction tools, and the reference genome. For any circRNA, if no best transcript is available, the corresponding circRNA was declared as “other” type. In the best transcript, if there was no exon within the circRNA boundary and an intron is contained within the circRNA boundary, we defined that circRNA as intronic. When there were some exons in the best transcript within the circRNA boundary, and the first and the last exon contained the start and end positions of the circRNA, respectively, we defined that circRNA as exonic, while the circRNA which was neither exonic nor intronic was declared as “other” type.

Let *O* be the output from the circRNA prediction tool and *T*_best_ be the best transcript for the *i*^th^ circRNA. Some variables were defined as

(1) $$\begin{array}{c} \text{Start}=\left\{\begin{array}{c} 1,\text{circRNA start position}\geq \text{start of }1^{\text{st}}\text{ exon of best transcript within circRNA boundary},\\ 0,\text{ otherwise}, \end{array}\right.\\ \text{End}=\left\{\begin{array}{c} 1,\text{circRNA end position}\leq \text{end of last exon of best transcript within circRNA boundary},\\ 0,\text{ otherwise}. \end{array}\right. \end{array}$$

For the *i*^th^ circRNA from *O*, the circRNA classification and sequence extraction were done using either of the following cases:

**Case 3 .**

**Figure 4:**
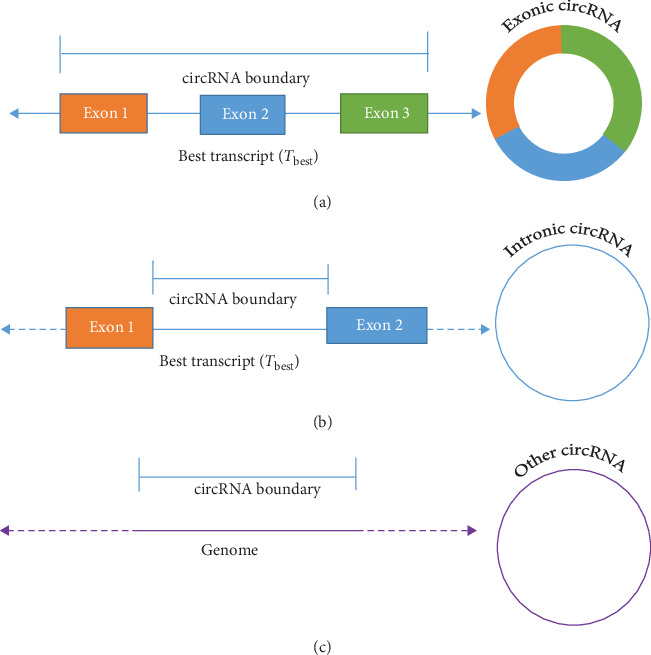
circRNA sequence extraction. (a) The circRNA is exonic, and its sequence is composed of combining the sequences of the three exons. (b) The circRNA is intronic, and its sequence consists of one intron sequence. (c) The circRNA is other type, and its sequence is the genomic sequence from start to end of the circRNA.

If start = 1 and end = 1 (Figure 4(a)), the circRNA is exonic, and the sequence is composed of the exons from *T*_best_ within the circRNA boundary (Figure 4(a)).

**Case 4 .**

If there are no exons and only one intron in *T*_best_ within the circRNA boundary, the circRNA is intronic. The sequence is composed of one intron from *T*_best_ (Figure 4(b)).

**Case 5 .**

If case 1 and case 2 are not satisfied, the circRNA is other type. The sequence is composed of a genomic sequence from start to end of the circRNA (Figure 4(c)).

## 3. Results

### 3.1. Extraction of Transcript Data and Full-Length circRNA Sequences

We have extracted the full-length circRNA sequences for the circRNAs downloaded from three databases, circbase, circRNAdb, and plantcircbase. For circbase and circRNAdb, only the human circRNAs have been used, and for plantcircbase, plant circRNAs have been used.

We have extracted the transcript data from the gene annotation file. Using the transcript data and the output of the circRNA prediction tools, we have created the circRNA classification file which contains the circRNA classification and all the required information for getting the full-length circRNA sequences. Using the start and end positions of circRNAs obtained from the circRNA prediction tool, we have extracted the genomic sequence from the reference genome. Finally, using the circRNA classification information and the genomic sequence, we have extracted the full-length circRNA sequences. The transcript data, circRNA classification, and full-length circRNA sequences are available at the supplementary Tables S1-S13, Tables S14-S28, and Tables S29-S43, respectively. The supplementary Tables S14-S28 (circRNA classification tables) have a total of 15 columns, and these columns represent, respectively, (1) circRNA ID, (2) chromosome, (3) circRNA start position, (4) circRNA end position, (5) circRNA strand, (6) circRNA length (7) circRNA type, (8) number of exons, (9) exon sizes, (10) exon offsets (start of each exon), (11) best transcript, (12) transcript strand, (13) transcript start, (14) transcript end, and (15) host gene.

### 3.2. Distribution of the circRNAs

**Figure 5:**
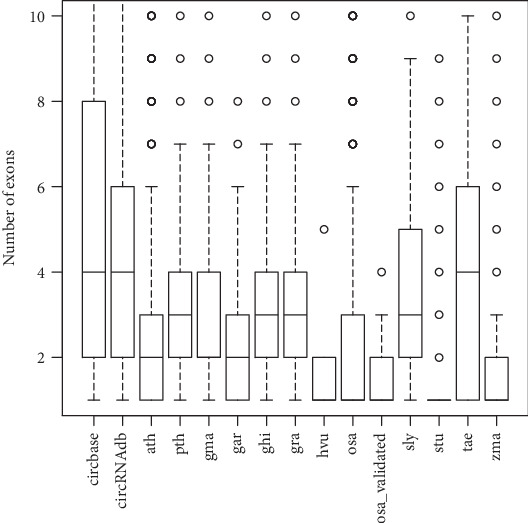
Boxplot of the number of exons for exonic circRNAs. Lower part of the box indicates *Q*_1_ (1^st^ quartile) which means 25% values of the exons are ≤*Q*_1_, middle part of the box indicates *Q*_2_(median) which means 50% values of the exons are ≤*Q*_2_, and the upper part of the box represent *Q*_3_ (3^rd^ quartile) which means 75% values of the exons are ≤*Q*_3_. The bars below and above the dashed line represent, respectively the minimum and maximum values.

In circbase, there are a total of 92375 human circRNAs; the extracted circRNA sequences by FcircSEC are 93.39% exonic, 0.75% are intronic, and 5.86% are others, while in circRNAdb, out of 32914 circRNAs, 99.32% are exonic, 0.02% are intronic, and 0.66% are others. Among the 67 experimentally validated circRNAs from plantcircbase, 62.69% are exonic and 37.31% are others, but no intronic is found. The classes of circRNAs for all other species are provided in Table 2. Again the distribution of the number of exons for the full-length exonic circRNAs is given in Figure 5. From Figure 5, we can observe that the median number of exons for most of the species is between 2 and 4.

### 3.3. Matched Sequences between Databases and FcircSEC

Since FcircSEC requires the chromosome name, start and end positions, and strand of each circRNAs as input, we have taken this information for each circRNA from the databases and extracted the full-length circRNA sequences using FcircSEC. Then, we have compared the sequences extracted by FcircSEC with those provided in the databases. During analysis, a sequence is matched if the whole sequence extracted by FcircSEC and the one provided in the database are identical (100%) and unmatched otherwise. We have calculated the proportion of matched sequences between FcircSEC and the three databases circbase, circRNAdb, and plantcircbase.  Table 3 lists the proportion of matched sequences.

In circbase and circRNAdb, there are a total of 92375 and 32914 full-length human circRNA sequences, respectively. We have extracted these circRNA sequences by FcircSEC and compared them with those of the databases. From Table 3, we can see that 95.1% and 98.9% of the sequences extracted by FcircSEC are exactly matched with circbase and circRNAdb, respectively. In plantcircbase, there are 67 (out of 95143) experimentally validated full-length circRNA sequences. We have extracted these 67 circRNA sequences by FcircSEC and found that all are exactly matched with the databases. We have also extracted the full-length sequences for the rest of the 95076 circRNAs (available in Supplementary Table S31-S43).

### 3.4. Comparison of FcircSEC with Alternative Methods

There are mainly four available tools for extracting full-length circRNA sequences: CIRI-full, FUCHS, circtools, and CircPrimer. Different methods depend on different prediction tools; for example, CIRI-full is dependent on CIRI, FUCHS is dependent on DCC, while CircPrimer and FcircSEC are not dependent on any prediction tools. Even some methods need RNA-seq data while others do not. As a result, performance of these methods is incomparable. Therefore, we have compared FcircSEC with the alternative methods in terms of application, limitation, etc. in Table 4.

From Table 4, we can see that CIRI-full takes the output of CIRI only as input, and RNA-seq data is needed to get the full-length sequence. It is not applicable if the lengths of all the reads in the RNA-seq data are not equal and if the annotation file is in gff format. Only the users of CIRI can use this tool for getting the full-length sequence. FUCHS and circtools take the output of DCC as input, and RNA-seq data is also needed to reconstruct the sequence. Both the tools are not applicable for short reads and cannot provide the full-length sequence directly. For both the tools, other software is needed to reconstruct the sequence. Both the tools are applicable for the users of DCC only. CircPrimer, although developed for designing primers, can extract the full-length sequences. But it is applicable for human circRNA only. FcircSEC can take the output of the state-of-the-art circRNA prediction tools as input. As RNA-seq data is not needed, there is no restriction in sequencing read lengths in using FcircSEC. It can take the annotation file in either gff or gtf format and is useful for human and other species. It can directly provide the full-length sequences. It can also classify circRNAs as three types (exonic, intronic, and others) while other methods cannot. The only limitation of FcircSEC is that it does not provide any information on splice sites within the circRNA sequence. In summary, we can say that FcircSEC has advantages over the existing methods.

## 4. Discussion

There are several circRNA prediction tools, but for only two tools CIRI and DCC, there is an existing method (CIRI-full and FUCHS) for getting the full-length sequences. For the users of other circRNA prediction tools (except CIRI and DCC), there are no existing tools for getting the full-length sequences. Although our method depends on the gene annotation information only, it will be a useful tool for the users who are interested in using the circRNA prediction tools other than CIRI and DCC.

CIRI-full and FUCHS can take the output of CIRI and DCC, respectively, as input, and hence, CIRI-full and FUCHS are applicable for the users of CIRI and DCC, respectively. circtools is also useful for DCC users as it uses the FUCHS module for circRNA sequence reconstruction. CircPrimer is applicable for human circRNAs only. Our method FcircSEC depends on the output of circRNA prediction tools, annotation information, and reference genome. FcircSEC can take the output of state-of-the-art circRNA prediction tools as input and, therefore, is applicable for almost all users of circRNA prediction tools.

Our method can extract the full-length circRNA sequence using the output of the existing circRNA prediction tools. We assume that the results of the existing circRNA prediction tools are correct, and we have not applied any filtering steps to detect the false positives. Again, within the circRNA boundary, we find a matching of the start of the first and the end of the last exons of the best transcript with the circRNA start and end positions. We assume that the circRNA contains all the intermediate exons, and we combine all the exons as a full-length circRNA. That is, we have not skipped any exons. This strategy is also used in CIRCexplorer.

FcircSEC does not take into account investigating the presence of the splice site within the circRNA sequence. For exonic circRNA, it combines all exons within the circRNA boundary to construct the full-length sequence. For the intronic and other types, it assumes that circRNAs are not spliced. By searching the databases circbase and circRNAdb, we have found that in almost all cases, the circRNA combines all exons. Besides, RNA-seq data is needed to examine the presence of a splice site within circRNAs. This is beyond the scope of the current work as FcircSEC is based on annotation information and does not take sequencing reads into account. This is the limitation of FcircSEC. We will try to overcome this limitation in the next version of the package.

Overall, the full-length sequence extraction is crucial in circRNA research. After predicting the candidate circRNAs, all the downstream analyses depend on the circRNA sequences. Therefore, FcircSEC can play an important role through extracting full-length circRNA sequences in identifying important circRNA biomarkers.

## 5. Conclusions

A number of methods are available in the literature for predicting the circRNA sequences. But only a limited number of methods are available for extracting full-length circRNA sequences. In this paper, we have developed an R package FcircSEC for extracting full-length circRNA sequences using the output of most of the popular circRNA prediction tools. The results of FcircSEC are consistent with the published circRNA databases and give more information that are not available in the public databases. Moreover, as for the users of the circRNA prediction tools other than CIRI and DCC, as there is no full-length circRNA sequence extraction method, FcircSEC can be a good choice for them. The R package FcircSEC is freely available at http://hpcc.siat.ac.cn/FcircSEC/Home.html.

## Figures and Tables

**Table 1:** Genome versions across species.

Species/database name	Genome/annotation version	Web links
circbase and circRNAdb (human)	hg19	http://hgdownload.soe.ucsc.edu/downloads.html#human http://202.195.183.4:8000/circrnadb/resources.php
Arabidopsis thaliana	TAIR10.38	ftp://ftp.ensemblgenomes.org/pub/plants/release-38/
Poncirus trifoliata	Citrus_clementina_v1.0	https://ftp.ncbi.nih.gov/genomes/refseq/plant/Citrus_clementina/
Glycine max	Glycine_max_v2.0.38	ftp://ftp.ensemblgenomes.org/pub/plants/release-38/
Gossypium arboreum	Gossypium_arboreum_v1.0	https://ftp.ncbi.nih.gov/genomes/refseq/plant/Gossypium_arboreum/
Gossypium hirsutum	NBI_Gossypium_hirsutum_v1.1	https://www.cottongen.org/data/download/genome_NBI_AD1/
Gossypium raimondii	Graimondii_221	ftp://ftp.jgi-psf.org/pub/compgen/phytozome/v9.0/Graimondii/
Hordeum vulgare	Hv_IBSC_PGSB_v2.38	ftp://ftp.ensemblgenomes.org/pub/plants/release-38/
Oryza sativa	IRGSP-1.0.38	ftp://ftp.ensemblgenomes.org/pub/plants/release-38/
Solanum lycopersicum	SL2.50.38	ftp://ftp.ensemblgenomes.org/pub/plants/release-38/
Solanum tuberosum	SolTub_3.0.38	ftp://ftp.ensemblgenomes.org/pub/plants/release-38/
Triticum aestivum	IWGSC1.0+popseq.29	ftp://ftp.ensemblgenomes.org/pub/plants/release-29
Zea mays	AGPv4.38	ftp://ftp.ensemblgenomes.org/pub/plants/release-38/

**Table 2:** Different types of circRNAs classified by FcircSEC.

Species/database name	circRNA types			Total
	Exonic (%)	Intronic (%)	Others (%)	
circbase	86267 (93.39)	695(0.75)	5413 (5.86)	92375
circRNAdb	32690 (99.32)	7 (0.02)	217 (0.66)	32914
Plantcircbase				
Arabidopsis thaliana	26643 (68.42)	1944 (4.99)	10351 (26.58)	38938
Poncirus trifoliata	242 (43.53)	5 (0.90)	309 (55.58)	556
Glycine max	2621 (49.24)	2086 (39.19)	616 (11.57)	5323
Gossypium arboreum	138 (13.41)	59 (5.73)	832 (80.86)	1029
Gossypium hirsutum	231 (46.29)	11 (2.20)	257 (51.50)	499
Gossypium raimondii	1231 (83.29)	10 (0.68)	237 (16.04)	1478
Hordeum vulgare	18 (46.15)	1 (2.56)	20 (51.28)	39
Oryza sativa	21849 (54.20)	9785 (24.27)	8677 (21.53)	40311
Oryza sativa (validated)	42 (62.69)	0 (0)	25 (37.31)	67
Solanum lycopersicum	1063 (55.83)	41 (2.15)	800 (42.02)	1904
Solanum tuberosum	584 (33.80)	3 (0.17)	1141 (66.03)	1728
Triticum aestivum	14 (15.91)	2 (2.27)	72 (81.82)	88
Zea mays	671 (20.72)	5 (0.15)	2562 (79.12)	3238

**Table 3:** Proportion of matched sequences between databases and FcircSEC.

Database	Species	Total circRNAs	No. of matched sequences	No. of unmatched sequences	Matched percentage
circbase	Homo sapiens	92375	87840	4535	95.1%
circRNAdb	Homo sapiens	32914	32538	376	98.9%
plantcircbase	Oryza sativa (validated)	67	67	0	100%

**Table 4:** Comparison of FcircSEC with alternative methods.

Method	Prediction tools whose output is taken as input	Is RNA-seq data needed?	Classify circRNAs?	Limitation	Applicability
CIRI-full	CIRI	Yes	No	Not applicable for unequal read lengths in the RNA-seq data and for the annotation file in gff format	Applicable for the users of CIRI
FUCHS	DCC	Yes	No	Not applicable for short reads and cannot provide full-length circRNA sequence directly	Applicable for the users of DCC
circtools	DCC	Yes	No	Not applicable for short reads and cannot provide full-length circRNA sequence directly	Applicable for the users of DCC
CircPrimer	State-of-the-art circRNA prediction tools	No	No	Not applicable for other than human circRNAs and does not yield any information on splice sites within the circRNA sequence	Applicable for human circRNAs only
FcircSEC	State-of-the-art circRNA prediction tools	No	Yes	Does not yield any information on splice sites within the circRNA sequence	Applicable for almost all users of circRNA prediction tools

## Data Availability

For circbase and circRNAdb, the annotation file is downloaded from circRNAdb (http://202.195.183.4:8000/circrnadb/resources.php) and the reference genome hg19 is downloaded from UCSC (http://hgdownload.soe.ucsc.edu/downloads.html#human). “Arabidopsis thaliana” annotation version TAIR10.38, “Glycine max” annotation version Glycine_max_v2.0.38, “Hordeum vulgare” annotation version Hv_IBSC_PGSB_v2.38, “Oryza sativa” annotation version IRGSP-1.0.38, “Solanum lycopersicum” annotation version SL2.50.38, “Solanum tuberosum” annotation version SolTub_3.0.38, “Triticum aestivum” annotation version IWGSC1.0+popseq.29, “Zea mays” annotation version AGPv4.38 and their reference genomes are downloaded from ftp://ftp.ensemblgenomes.org/pub/plants/.

## Supplementary Materials

Supplementary Materials Supplementary Materials. Supplementary Tables S1–S13: transcript data for different species are given. Supplementary Tables S14–S28: circRNA classification data for different species are provided. Supplementary Tables S29–S43: full-length circRNA sequences across species are supplied.
