# Supplementary material for: FcircSEC: An R Package for Full Length circRNA Sequence Extraction and Classification
Source: Int J Genomics. 2020 May 28;2020:9084901. doi: 10.1155/2020/9084901 (PMC7285417; doi:10.1155/2020/9084901)
Supplement: Supplementary Materials — Supplementary Tables S1–S13: transcript data for different species are given. Supplementary Tables S14–S28: circRNA classification data for different species are provided. Supplementary Tables S29–S43: full-length circRNA sequences across species are supplied. [file 9084901.f1.zip › Supplementary_tables_legend.docx]

**Legend of Supplementary Tables:**

| **Transcript data** | |
| --- | --- |
| TableS1 | Transcript data for circRNAdb and circbase database |
| TableS2 | Transcript data for Arabidopsis thaliana |
| TableS3 | Transcript data for C_clementina |
| TableS4 | Transcript data for Glycine max |
| TableS5 | Transcript data for Gossypium arboreum |
| TableS6 | Transcript data for Gossypium hirsutum |
| TableS7 | Transcript data for Gossypium raimondii |
| TableS8 | Transcript data for Hordeum vulgare |
| TableS9 | Transcript data for Oryza sativa |
| TableS10 | Transcript data for Solanum lycopersicum |
| TableS11 | Transcript data for Solanum tuberosum |
| TableS12 | Transcript data for Triticum aestivum |
| TableS13 | Transcript data for Zea mays |
| **circRNA classification** | |
| Table S14 | Circular RNA classification for circRNAdb database |
| Table S15 | Circular RNA classification for cirbase database |
| Table S16 | Circular RNA classification Arabidopsis thaliana |
| Table S17 | Circular RNA classification C_clementina |
| Table S18 | Circular RNA classification for Glycine max |
| Table S19 | Circular RNA classification for Gossypium arboreum |
| Table S20 | Circular RNA classification for Gossypium hirsutum |
| Table S21 | Circular RNA classification for Gossypium raimondii |
| Table S22 | Circular RNA classification for Hordeum vulgare |
| Table S23 | Circular RNA classification for Oryza sativa |
| Table S24 | Circular RNA classification for Oryza sativa (validated) |
| Table S25 | Circular RNA classification for Solanum lycopersicum |
| Table S26 | Circular RNA classification for Solanum tuberosum |
| Table S27 | Circular RNA classification for Triticum aestivum |
| Table S28 | Circular RNA classification Zea mays |
| **circRNA sequences** | |
| Table S29 | Circular RNA sequence for circRNAdb database |
| Table S30 | Circular RNA sequence for cirbase database |
| Table S31 | Circular RNA sequence for Arabidopsis thaliana |
| Table S32 | Circular RNA sequence for C_clementina |
| Table S33 | Circular RNA sequence for Glycine max |
| Table S34 | Circular RNA sequence for Gossypium arboreum |
| Table S35 | Circular RNA sequence for Gossypium hirsutum |
| Table S36 | Circular RNA sequence for Gossypium raimondii |
| Table S37 | Circular RNA sequence for Hordeum vulgare |
| Table S38 | Circular RNA sequence for Oryza sativa |
| Table S39 | Circular RNA sequence for Oryza sativa (validated) |
| Table S40 | Circular RNA sequence for Solanum lycopersicum |
| Table S41 | Circular RNA sequence for Solanum tuberosum |
| Table S42 | Circular RNA sequence for Triticum aestivum |
| Table S43 | Circular RNA sequence for Zea mays |

The supplementary Tables S14-S28 (circRNA classification Tables) have total 15 columns and these columns represent respectively (1) circRNA ID, (2) chromosome, (3) circRNA start position, (4) circRNA end position, (5) circRNA strand, (6) circRNA length (7) circRNA type, (8) Number of exons, (9) Exon sizes, (10) Exon offsets (start of each exon), (11) Best transcript, (12) Transcript strand, (13) Transcript start, (14) Transcript end, and (15) Host gene.
